# Implantable Dental Barrier Membranes as Regenerative Medicine in Dentistry: A Comprehensive Review

**DOI:** 10.1007/s13770-025-00704-1

**Published:** 2025-02-24

**Authors:** Siyuan Chen, Zhenzhen Wu, Ziqi Huang, Chao Liang, Sang Jin Lee

**Affiliations:** Biofunctional Materials, Division of Applied Oral Sciences and Community Dental Care, Faculty of Dentistry, The University of Hong Kong, 34 Hospital Road, Sai Ying Pun, Hong Kong SAR People’s Republic of China

**Keywords:** Dental membrane, Oral tissue regeneration, Periodontal disease, GTR/GBR, Oral bioscience

## Abstract

**Background:**

Periodontitis and bone loss in the maxillofacial and dental areas pose considerable challenges for both functional and aesthetic outcomes. To date, implantable dental barrier membranes, designed to prevent epithelial migration into defects and create a favorable environment for targeted cells, have garnered significant interest from researchers. Consequently, a variety of materials and fabrication methods have been explored in extensive research on regenerative dental barrier membranes.

**Methods:**

This review focuses on dental barrier membranes, summarizing the various biomaterials used in membrane manufacturing, fabrication methods, and state-of-the-art applications for dental tissue regeneration. Based on a discussion of the pros and cons of current membrane strategies, future research directions for improved membrane designs are proposed.

**Results and conclusion:**

To endow dental membranes with various biological properties that accommodate different clinical situations, numerous biomaterials and manufacturing methods have been proposed. These approaches provide theoretical support and hold promise for advancements in dental tissue regeneration.

## Introduction: overview of dental membrane use and applications in clinical cases

**Fig. 1:**
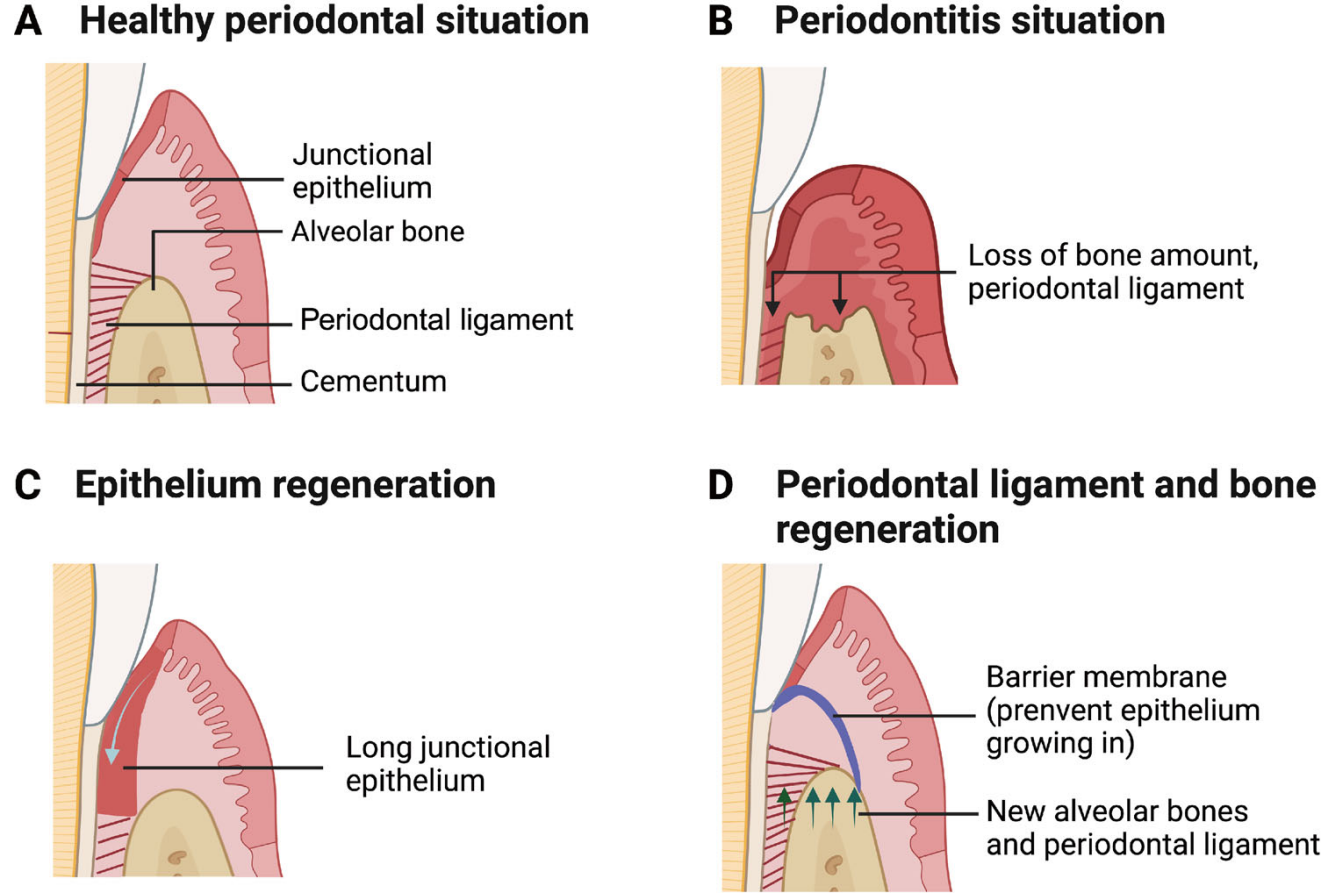
Different situations of periodontal tissues. **A** In a healthy periodontal situation, the junctional epithelium forms for sealing gingival sulcus. Beneath this, the periodontal ligament connects one end to the cementum and the other to the alveolar bone. **B** In the periodontitis situation, the alveolar bones and periodontal ligament are resorbed, leading to bone defect. **C** In normal reconstruction situations, the empty defect area is often occupied by epithelium cells due to its faster immigration speed, resulting in the long junctional epithelium connection. **D** Once the barrier membrane is positioned over the bone defect, it shelters the osteoblasts and periodontal ligament cells from epithelium occupation, securing periodontal tissue regeneration. (Created in https://BioRender.com)

Maxillofacial and alveolar tissue defects resulting from trauma, tumors, and periodontitis often pose functional and aesthetic challenges for patients [1]. In ideal periodontal reconstruction scenarios, an epithelial seal is re-established, accompanied by the deposition of new fiber cementum and the reconstruction of the alveolar bone, all connected by the functionally oriented insertion of the periodontal ligament [2]. The successful oseteanagenesis in maxillofacial areas also relies on the restoration of bone volume. However, at defect sites, the migration of epithelial cells often outpaces that of osteocytes and periodontal ligament cells [3], leading to premature occupation by the epithelium and insufficient tissue reconstruction [4] (Fig. 1).

To address this issue, inhibition membranes have been documented for use in guided tissue regeneration /guided bone regeneration (GTR/GBR) since the 1980s, with long-term follow-up studies demonstrating positive and comparable clinical outcomes [5]. Initially, these membranes were broadly categorized into resorbable and non-resorbable types. However, due to the necessity of secondary surgeries and the improper stiffness of non-resorbable membranes (some are so hard that they may contribute to soft tissue dehiscence), their practical applications have gradually diminished [6]. Further exploration in preclinical and clinical research has led to the design of ideal dental membranes, which consider key standards such as the PASS principle (P: primary closure, A: angiogenesis, S: space maintenance, S: stability) [7]. To achieve the desired effects and enhance multifunctionality, innovative materials and manufacturing methods are now being applied to GTR/GBR membranes.

**Fig. 2:**
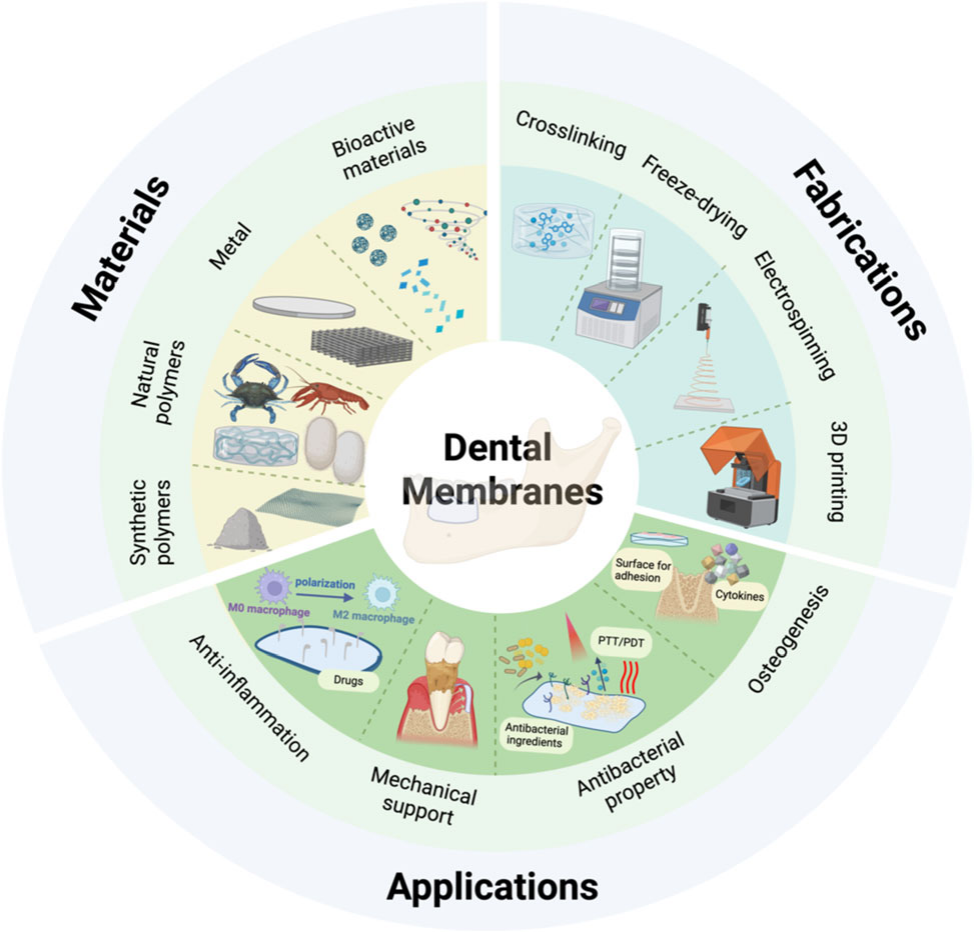
Schematic illustration of representative dental membrane designs. Some critical factors from fields of materials, fabrication and applications may define the success of membranes. The material is of great significance for bioactive and mechanical properties of membranes. Fabrication methods should be selected properly for membrane structures aiming at promoting cell adhesion, mechanical support and so on. The ideal dental membranes can be applied into different clinical situations for biological problem solving. (Created in https://BioRender.com)

This review focusing on three key points-materials, fabrications and application scenarios, covering the latest advancements in barrier membranes within the maxillofacial and alveolar fields (Fig. 2). Additionally, the pros and cons of different designs will be discussed, which could aid researchers and clinicians in integrating and translating these innovations into clinically applicable strategies more effectively. Finally, we will propose potential improvement directions for the future development of these dental membranes within the broader context of regenerative medicine. This not only reflects the current research limitations but also provides valuable insights for future investigations in this field.

## Membranes manufactured biomaterials for dental tissue engineering

### Synthetic polymers

#### Nonbiodegradable synthetic polymers

**Fig. 3:**
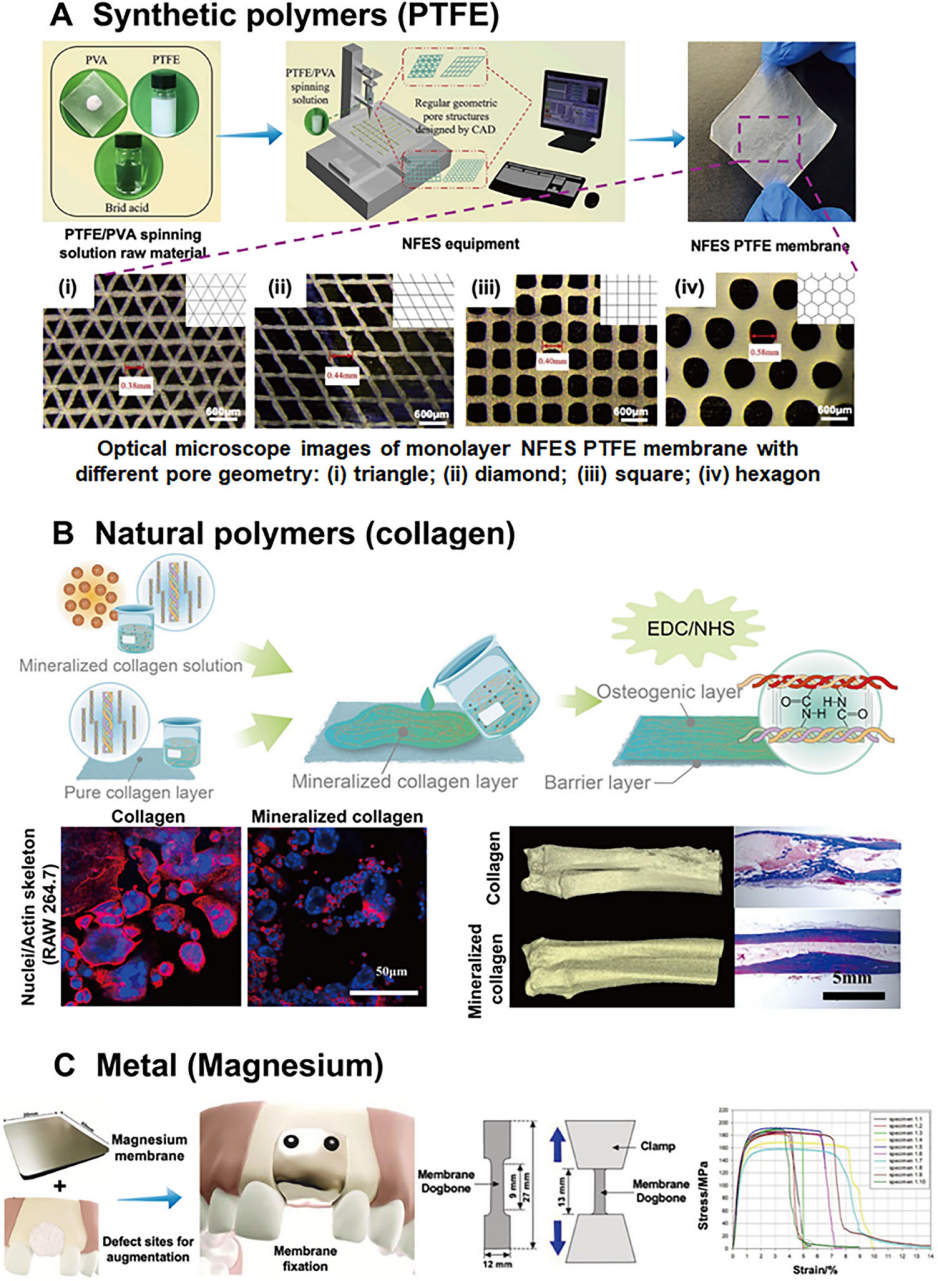
Different material composition of dental barrier membranes. **A** Design of a novel PTFE membrane with different geometric pore structures fabricated by near-field electrospinning for cell adhesion. Adapted with permission from Cheng et al. [9] Copyright (2020), Elsevier. **B** Schematic illustration and biological results of bilayer mineralized collagen/collagen membrane for enhanced osteogenesis and anti-inflammation in GBR applications. Adapted with permission from Peng et al. [37] Copyright (2023), Springer. **C** Design of biodegradable magnesium barrier membrane with mechanical property tests for dental surgery, which possesses superior stiffness for supporting the collapse defect areas. Adapted with permission from Rider et al. [84] Copyright (2022), Elsevier

Polytetrafluoroethylene (PTFE) is a material predominantly used for non-biodegradable membranes in the field of dental tissue engineering [8], particularly for GTR/GBR in the treatment of periodontal diseases, as shown in Fig. 3A [9]. The first example of GTR/GBR successfully employed non-resorbable membranes made of methylcellulose acetate (Millipore, Bedford, MA, USA). However, this membrane material was quickly replaced by expanded polytetrafluoroethylene (e-PTFE) due to its superior durability [10–13]. e-PTFE is a synthetic polymer composed of fluorine atoms linked to a long carbon backbone. It is classified as a bioinert material, known for its superior stability and biocompatibility [14, 15]. e-PTFE membranes have the ability to integrate with the bone and connective tissue at the edge of periodontal lesions while specifically blocking the migration of gingival and epithelial connective tissue cells. This property makes them widely used in clinical treatments [16–18]. However, because PTFE serves as a strong barrier between tissues, it can reduce blood flow and contribute to gingival dehiscence, which is associated with a high prevalence of early spontaneous gingivitis [15].

Recently, another compact form of this synthetic polymer, named dense PTFE (d-PTFE), was proposed and introduced to the market [19]. It has been reported that d-PTFE and e-PTFE membranes have similar clinical outcomes in vertical bone treatment and ridge augmentation [20]. However, the d-PTFE membrane is easier to remove than the e-PTFE membrane when flap elevation is required [21]. In this context, it has been suggested that d-PTFE be used in areas with significant ridge atrophy to prevent graft contamination in the event that the membrane is undesirably exposed [22].

#### Biodegradable synthetic polymers

The primary reason for developing bioabsorbable and biodegradable membranes is to eliminate the need for a second procedure to remove the membrane [23]. Aside from natural materials, the most studied and commonly used bioabsorbable polymers in clinical applications are aliphatic or aromatic polyesters and polysaccharides [24]. Membranes produced from aliphatic polyesters, such as polycaprolactone (PCL), polylactic acid (PLA), and poly (lactic-co-glycolic acid) (PLGA), are particularly prevalent in the dental field [25–27]. Aliphatic polyester membranes offer the advantage of adjustable mechanical properties and biodegradability, which can be tailored by varying the polymer composition [28]. Additionally, these synthetic polymers can be processed industrially, increasing the potential for reproducible production compared to natural materials [29]. Furthermore, it is easy to impregnate drugs and substances that promote tissue regeneration into these membranes. This property makes aliphatic polyesters becoming excellent substrate materials for GTR/GBR technology, as well as key components in dental composite membranes [30]. However, due to the difficulty in enhancing the mechanical properties of biodegradable polyesters alone, these synthetic membranes are often used in conjunction with bone substitutes or for minor tissue defects [29].

In recent years, numerous dental membranes made from biodegradable synthetic polymers have emerged in the field of dental tissue engineering. For instance, Liao et al*.* proposed a novel functionally graded membrane composed of nano-carbonated hydroxyapatite (HA, Ca_10_(PO_4_)_6_(OH)_2_) and PLGA in 2005 [31]. The design of this dental membrane is highly sophisticated; one side made from PLGA is smooth to prevent cell attachment, while the other side, composed of HA, is porous, effectively promoting cell proliferation [31]. In 2011, Bottino et al*.* fabricated a type of multilayer continuous GTR membrane made from poly(dl-lactide-co-ε-caprolactone) (PLCL) and PLA with gelatin loaded with nano-HA (n-HA) via electrospinning for the first time. This system was reported to maintain its mechanical, chemical, and physical properties over an extended period, achieving optimal effects in periodontal regeneration [32]. In 2017, Ke et al*.* utilized PCL and gelatin to produce as-spun nanofiber membranes through genipin crosslinking. Their study demonstrated that these engineered electrospun polymer/protein nanofibers could serve as promising candidates for efficient GBR membranes [33]. Furthermore, in 2021, Peng et al*.* developed a composite membrane based on PLGA with artemisinin. The excellent antibacterial and anti-inflammatory properties of this membrane suggest it could be an effective wound dressing for chronic wounds [34]. In conclusion, composite membranes made from biodegradable synthetic polymers have great potential to play a crucial role in dental tissue engineering, particularly for guided tissue and bone regeneration.

### Natural polymers

In addition to synthetic polymers, many natural polymers are also ideal materials for dental membrane fabrication due to their excellent biocompatibility and biodegradability. However, the mechanical properties, such as compressive strength, of most natural polymers are not particularly strong. Therefore, scientists often combine natural polymers with synthetic polymers to create composite materials for the manufacture of dental barrier membranes [29].

#### Collagen

Collagen is an indispensable component of the body that can be extracted from muscle, connective tissues, and bones [35, 36]. Besides maintaining the structure of cells and tissues, collagen also plays a significant role in promoting blood vessel formation, cell adhesion, tissue healing, and other biological processes (Fig. 3B). [37, 38]. The human body contains a variety of specialized cells that synthesize collagen, depending on its location. In connective tissue, osteoblasts are responsible for producing collagen, while fibroblasts are involved in the production of collagen in bones [39]. With minimal immunogenicity, intrinsic bioactivity, and biodegradability, collagen is the most prevalent extracellular matrix (ECM) protein, and collagen-based bioactive membranes are common choices in tissue engineering applications [40, 41]. To overcome the limitations of natural collagen materials, such as poor mechanical stability and rigidity, it is necessary to process the original collagen through decellularization, cross-linking, or sterilization to create effective dental barrier membranes [42, 43]. Several methods for cross-linking collagen have been reported, including physical cross-linking techniques such as ultraviolet irradiation and chemical methods using agents like genipin, carbodiimide, or glutaraldehyde. These methods have been shown to enhance stability and delay degradation successfully [18]. The successful development and use of resorbable collagen membranes for GBR treatment has been documented, with these membranes lasting up to six months in the human body [44]. However, the remaining chemical cross-linking agents may pose a serious risk for clinical use due to their potential toxicity, which can cause inflammation and interfere with cellular processes [45–47]. Additionally, although cross-linking can improve mechanical strength to some extent, the low rigidity of collagen remains a concern in clinical applications. Therefore, collagen membranes are more appropriate for use in the areas where high mechanical requirements are not needed [48].

#### Gelatin

Gelatin is a naturally occurring polymer derived from the hydrolytic breakdown of collagen protein, and its unique amino acid composition offers many health benefits compared to the original collagen [49–51]. Due to its excellent characteristics, such as superb biodegradability, ideal biocompatibility, low immunogenicity, abundance of resources, and low cost, gelatin has been widely employed in the drug delivery, dental tissue engineering, and hemostasis areas [52–55]. However, the clinical application of gelatin is limited by its excessively rapid rate of degradation, incompatibility in high humidity conditions, and weak mechanical properties [56]. Various cross-linking techniques, including physical, chemical, and biological methods, have been applied to enhance the mechanical properties of gelatin and control its degradation rate. Although these techniques can significantly improve mechanical properties, cross-linked gelatin still has several shortcomings, such as the cytotoxicity of cross-linking chemical reagents and inadequate capacity for bone regeneration and vascularization [57]. Furthermore, it has been shown that cross-linked gelatin possesses an extremely low Young's modulus yet high elastic strength in moist conditions [58]. Therefore, combining gelatin with other biodegradable synthetic polymers, rather than using pure gelatin for GTR/GBR membranes, is considered a common solution to effectively address these drawbacks in the fields of tissue engineering and regenerative medicine [59].

#### Chitosan (CS)

In order to overcome the mechanical performance limitations of collagen and gelatin, researchers are committed to finding alternative natural polymers for GBR/GTR membranes to enhance their rigidity, particularly in areas subject to high mechanical stress [60, 61]. Among various materials, CS, a deacetylated form of chitin found in crustacean exoskeletons, has been demonstrated to be a desirable candidate for GTR and GBR membranes due to its favorable biocompatibility, suitable degradation rate, excellent antibacterial properties, flexibility in moist conditions, lack of antigenicity, low cost, and potential for wound healing [62–65]. Similar to collagen-based membranes, the most effective way to enhance the mechanical strength of CS and slow its degradation rate is through chemical cross-linking [66]. One study revealed that after 16 weeks of *in vitro* testing, genipin-cross-linked CS electrospun membranes showed only 22% degradation, a significantly slower rate than that of non-cross-linked mats, which experienced 34% degradation. Furthermore, the cross-linked membranes exhibited high ultimate tensile strength, nearly 165% greater than that of the non-cross-linked membranes [67]. Coupled with the inherent superior antibacterial activity of CS, these findings suggest that genipin-cross-linked CS membranes may have the potential to meet clinical requirements for GTR/GBR applications in dentistry [68–70]. However, there is a lack of human clinical trial research on these membranes, making it difficult to determine their long-term safety and effectiveness [71]. Additionally, CS has not yet been approved by the American Food and Drug Administration as a clinical treatment ingredient [72]. Therefore, the feasibility and viability of CS-based GTR/GBR membranes still require further verification for clinical applications.

#### Silk fibroin (SF)

SF is a collagen-like fiber derived from silk cocoons or spider silk, known for its exceptional mechanical properties and excellent permeability of water vapor and oxygen [73]. *In vitro* studies have shown that SF elicits minimal immunological reactions and promotes superior cell adhesion and proliferation across various cell types. Simultaneously, *in vivo* experiments indicate that SF films provoke a low inflammatory response, and the degradation products of SF are amino acids, which are non-toxic and harmless to organisms [73–75]. Equipped with these unique properties and low cost, SF has emerged as a promising material for tissue and bone regeneration in dentistry. In recent years, numerous scientists have reported on the use of SF as a scaffold for prosthetic skin, angiogenesis, wound dressings, and bone graft materials in tissue engineering. SF-based GTR/GBR dental membranes have also become a significant area of research. For instance, Song et al*.* dissolved SF in a CaCl_2_ solution and cast SF nanofiber membranes after dialysis purification. It was reported that rabbits with calvarial defects exhibited significantly higher bone regeneration after grafting this type of SF nanofiber membrane, with complete repair of the defect observed after 12 weeks [76]. Additionally, to assess the viability and effectiveness of using SF for potential GBR membranes, Yoo et al*.* conducted research examining the cellular responses of osteoblast-like MG-63 to SF. The results demonstrated that the cell attachment abilities of SF are comparable to those of other commercially available membranes [77]. Furthermore, it was indicated that SF possesses exceptional strength and toughness, providing space for bone ingrowth while preventing membrane collapse [78]. Overall, these findings collectively provide compelling evidence that SF membranes may have valuable applications as GBR/GTR barrier membranes.

### Metal

#### Titanium (Ti)

Non-resorbable and porous Ti mesh was first employed in 1969 by Boyne et al*.* for bone restorations and now is particularly useful in therapeutic and clinical applications [79]. Due to its robust construction, Ti mesh provides significantly better space management, collapse avoidance, and surgical malleability compared to other materials commonly used for GTR/GBR membranes in dental tissue engineering. Furthermore, Ti possesses several enhanced physical properties, including lightweight design, exceptional strength and stiffness, high durability, and the ability to withstand elevated temperatures, increasing its viability for various surgical applications [80]. Due to its low density and notable flexibility, Ti membranes can bend and adapt to the shape of bone defects or ridges [81]. Compared with the traditional e-PTFE and d-PTFE dental membranes, the macroporosity of Ti meshes is also beneficial for blood and nutrition supply management, as well as enhancing tissue integration in the area of wounds [82].

However, Ti is unsuitable for some patients due to the requirement for complex secondary removal surgery [83]. Additionally, the sharp edges of the Ti mesh may complicate the removal procedure and result in microbial contamination in the surgical field [80]. Despite these challenges and limitations, Ti mesh remains a viable option for guided bone and tissue regeneration treatments due to its excellent biocompatibility and potential for improved tissue integration.

#### Magnesium (Mg)

Although Ti-reinforced membranes can provide adequate mechanical protection for covered bone defects compared to simple polymer membranes, an inevitable drawback is that they require removal through a second surgery [84]. Considering the increasing demands for both adequate mechanical strength and resorbability during the bone grafting process in recent years, biodegradable metals such as Mg, zinc (Zn), and iron (Fe) represent a novel option for scientists and clinicians. According to previous reports, Mg membranes have numerous beneficial properties, including excellent mechanical stability (Fig. 3C) [84], complete degradability, and a biocompatible surface that promotes the migration and adhesion of human gingival fibroblasts [85]. It has been shown that pure Mg membranes have a lower elastic modulus and extremely higher tensile strength (183 ± 10.7 MPa) compared to SF membranes (8.54 ± 0.63 MPa) and commercial biodegradable membranes (11.72—14.50 MPa), according to mechanical tests conducted by White et al*.* This strength could prevent collapse and significantly promote regeneration of vertical defects [84, 86, 87]. In the same study, the results of the tear resistance test demonstrated that the Mg membrane exhibited superior tear resistance, remaining intact even when the Ti membrane fixation screw failed [84]. Moreover, the fully absorbable and biodegradable properties of Mg membranes, without any toxic residues, offer outstanding clinical potential for guided bone and tissue regeneration [88–91]. Mg can degrade into Mg ions (Mg^2+^) and hydrogen gas under the action of enzymes, both of which are harmless and can be reutilized by the body [92]. Mg^2+^ is naturally present in nearly every human organism and is involved in numerous crucial physiological processes [93, 94]. It has been shown that there are effective mechanisms for excreting Mg^2+^ through the kidneys and intestines when intake is below the recommended level (280 mg per day), indicating that Mg membranes possess convincing non-toxicity and biosafety for dental regenerative medicine [94–96]. Furthermore, the surface of the Mg membrane promotes stronger cell adhesion and migration, suggesting that it can help reconstruct the osseous structure while simultaneously protecting the papilla and soft tissue above [96]. Additionally, *in vitro* studies have revealed that exposure to Mg enhances cell proliferation and the expression of osteogenic markers [97, 98]. Overall, Mg is suggested as an optimal candidate material for dental barrier membranes based on both mechanical and biological properties.

#### Zn

Zn, like Mg, is a versatile metal that has been widely utilized in various biomedical applications in recent years due to its excellent mechanical, biodegradable, and biocompatible properties [99]. As an essential trace element in the human body, Zn not only interacts with several chemical ligands but also plays a pivotal role in fundamental biological processes such as gene expression, signal transduction, and apoptotic regulation [100]. More importantly, previous studies have demonstrated that Zn ions (Zn^2+^) can accelerate the growth and differentiation of osteogenic cells while suppressing the activity of bone resorption cells, thereby stimulating the regeneration of new bone [101–103]. Additionally, Zn has ability to enhance cellular protein production, activates aminoacyl-Transfer Ribonucleic Acid synthetase in osteoblastic cells, and helps maintain bone mass, all of which are essential for the development and mineralization of bone tissues [102, 104]. Moreover, pure Zn membranes exhibit a relatively suitable degradation rate and do not produce a large number of degradation products during degradation tests [99, 105]. Another study indicated that using a mineralized membrane with Zn phosphate could reduce inflammation caused by membrane exposure and exhibit antibacterial activity, which is beneficial for the restoration of soft and hard tissues [106]. Therefore, the excellent degradation behavior and ability to inhibit oral bacterial colonization make Zn one of the most promising materials for dental implants.

### Incorporated materials in membrane

#### Addition of bioceramics

Over the past two decades, significant progress has been made in the field of bioceramics used for regenerative medicine in dentistry [107]. These materials exhibit superior bioactivity and biocompatibility and have been widely utilized as additives to enhance the mechanical performance and tissue regeneration of dental membranes [108–110]. Among the various types of bioceramics, calcium phosphate has been shown to play critical roles in cell adhesion, tissue management, cellular signaling, and bone regeneration, especially for possessing osteoinductive and osteoconductive properties. Calcium ions promote bone formation and maturation through calcification, while phosphate ions are involved in the development and activation of osteoblasts [18, 111, 112]. According to the literature, the calcium phosphate used in dentistry is mainly divided into two categories: HA and biphasic beta-tricalcium phosphate (β-TCP). The chemical composition of HA is very close to that of the mineral phase found in natural teeth, making it a common additive in GTR/GBR membranes [113–115]. However, ordinary HA lacks significant antibacterial properties [116]. Recently, various modifications of HA have been explored by substituting calcium ions with antimicrobial divalent metal ions. For example, Tang et al*.* proposed a polycaprolactone/cobalt-substituted HA membrane for bone tissue engineering, demonstrating that the addition of cobalt-HA powder significantly promotes cell proliferation and provides superior antibacterial and anti-inflammatory effects [115]. Another study by Sherif Elbasuney et al*.* indicated that silver (Ag)-doped HA can reduce the overall viable bacterial count by completely lysing bacterial cells and causing cell deformity, making it a promising material for dental regenerative medicine and tissue engineering [117]. Additionally, n-HA is another form of this material commonly used for bone reinforcement. A study on composite membranes made of n-HA and CS showed that the n-HA/ CS asymmetric membranes had greater break elongation and tensile strength compared to pure CS membranes under wet conditions [118]. Another study on n-HA/polyvinyl alcohol (PVA) composites illustrated that n-HA nanoparticles could enhance the Young’s modulus of membranes in mechanical property tests, and the surface of the composite membranes showed promising functionality for osteogenic cell adhesion and proliferation when n-HA concentration was no more than 20 wt% [119]. Overall, it has been demonstrated that membranes containing HA significantly improve bone augmentation at the insertion site [18].

As another member of the calcium phosphate family, β-TCP shares similar characteristics with HA, including excellent cell adhesion, osteoconductivity, and biodegradability [120–122]. To enhance its mechanical performance and degradation rate, β-TCP is often combined with other biopolymers such as collagen, PCL, and PLGA. In 2016, Won et al*.* combined β-TCP with PLGA and PCL, fabricating a printed PCL/PLGA/β-TCP membrane for guided bone formation in a beagle implant model. Compared to commercial collagen membranes, the proposed membrane enhanced hard tissue regeneration *in vivo* without the need for bone replacement and also promoted mesenchymal stem cell osteogenic differentiation and infiltration *in vitro* [123].

In addition to calcium phosphate materials, another common bioceramic with superior osteogenic qualities is bioactive glass, which is frequently utilized to promote dental tissue regeneration. Generally, the primary component of bioactive glass is SiO_2_, which is an amorphous substance with excellent biodegradability [124–126]. Under these conditions, bioactive glass can release calcium and silicate ions by adjusting and controlling the degradation rate, transforming into HA-like materials, which ultimately increases osteoblast activity and their connection to bone [127]. Furthermore, recent research has suggested that bioactive glass can stimulate vascularization, which is beneficial not only for bone regeneration but also for soft tissue wound repair [128–130]. Additionally, bioactive glass has been shown to have positive effects on osteochondral tissue engineering by promoting the production of neocartilage during the *in vitro* cultivation of chondrocyte-laden hydrogels [131]. However, the inherent brittleness of bioactive glass is a significant limitation that restricts its clinical application to a great extent [132]. Therefore, future studies must focus on mitigating this brittleness through the development of innovative scaffolds while simultaneously harnessing the advantageous characteristics of bioactive glass, especially for load-bearing bone restoration [124].

#### Addition of functional substance

The ability of membranes to promote bone regeneration can be significantly enhanced by adding functional substances, including growth factors, drugs, or other components that can induce biological effects in the damaged area [18]. Common growth factors utilized in dental implants include transforming growth factor β (TGF-β), vascular endothelial growth factor (VEGF), bone morphogenic proteins (BMPs), platelet-derived growth factor (PDGF), insulin-like growth factors (IGF), and fibroblast growth factor (FGF-2) [133–135]. Among these proteins, the transforming TGF-β family plays a prominent role in regenerative medicine, particularly in soft tissue wound healing. Members of the TGF-β family, including growth and differentiation factors, regulate a wide range of biological processes, such as cell migration, survival, and differentiation. These factors have become promising additives in dental membranes to promote bone and vascular tissue regeneration in oral and maxillofacial reconstruction for both preclinical and clinical applications [136, 137]. For instance, Gruber et al*.* first incorporated platelet-rich fibrin-derived TGF-β into collagen membranes and Ti surfaces through activity absorption, demonstrating significant resistance to vigorous washing [138]. It has also been found that TGF-β, BMPs, and other growth factors can adsorb to collagen and work synergistically to improve growth factor binding and release, achieving sustained delivery of growth factors to the bone defect area in dental tissue engineering. However, the specific mechanisms still need to be confirmed through further studies [139, 140].

Moreover, as previously mentioned, some metals, such as Zn and Mg, positively affect bone and vascular regeneration. Therefore, incorporating metal nanoparticles is also a crucial strategy for GTR/GBR membrane fabrication, enhancing antibacterial properties simultaneously. In 2018, Kwon et al*.* developed a CS/polyurethane (CSP) nanofibrous membrane integrated with Ag nanoparticles using electrospinning. This composite membrane exhibited excellent biocompatibility and antimicrobial performance against *Porphyromonas gingivalis*. Notably, the Ag/CSP membrane maintained antibacterial activity while containing only trace amounts of Ag nanoparticles, indicating that it is possible to achieve a balance between cytotoxicity and antibacterial properties by regulating the content of Ag nanoparticles [141]. In 2020, Wu et al*.* reported a biodegradable Mg oxide nanoparticle (nMgO)-loaded PLA/gelatin nanofibrous membrane. Their findings illustrated that the addition of nMgO dramatically promoted the osteogenesis of the membranes, as seen in both *in vitro* and *in vivo* experiments, which the authors attributed to the Mg^2+^ released from nMgO [142].

Additionally, metal–organic frameworks (MOFs) have emerged as promising materials in biomedical engineering. MOFs are porous hybrid structures composed of various organic ligands connected with metallic coordination nodes coordinately, exhibiting significant variability in physicochemical properties and pore volumes due to their customizable compositions and architectures [143]. Zeolitic imidazolate framework-8 (ZIF-8) has demonstrated significant biomolecule loading and release efficiency, along with reasonable stability and biocompatibility, making it potentially impactful in dental regenerative medicine and tissue engineering [144]. In 2024, Dai et al*.* proposed a multifunctional Janus membrane incorporated with both HA and methylene blue @ ZIF-8 nanoparticles for antimicrobial and guided tissue regeneration applications. Researchers constructed a periodontal bone defect model in rats, and the results showed that the proposed membranes possessed excellent osteogenic qualities, barrier performance, and superior antibacterial properties, indicating a wide range of uses in managing periodontal bone abnormalities [145].

## Fabrication methods for dental membranes

### Conventional manufacturing methods

Driven by the promising characteristics offered by guided tissue and bone regeneration, various manufacturing methods have been proposed to create dental barrier membranes in diverse forms. The conventional fabrication techniques include freeze-drying [146], emulsion templating [147], solution casting [148], and electrospinning [141]. It is also feasible to combine these manufacturing procedures to optimize membrane fabrication [149].

**Fig. 4:**
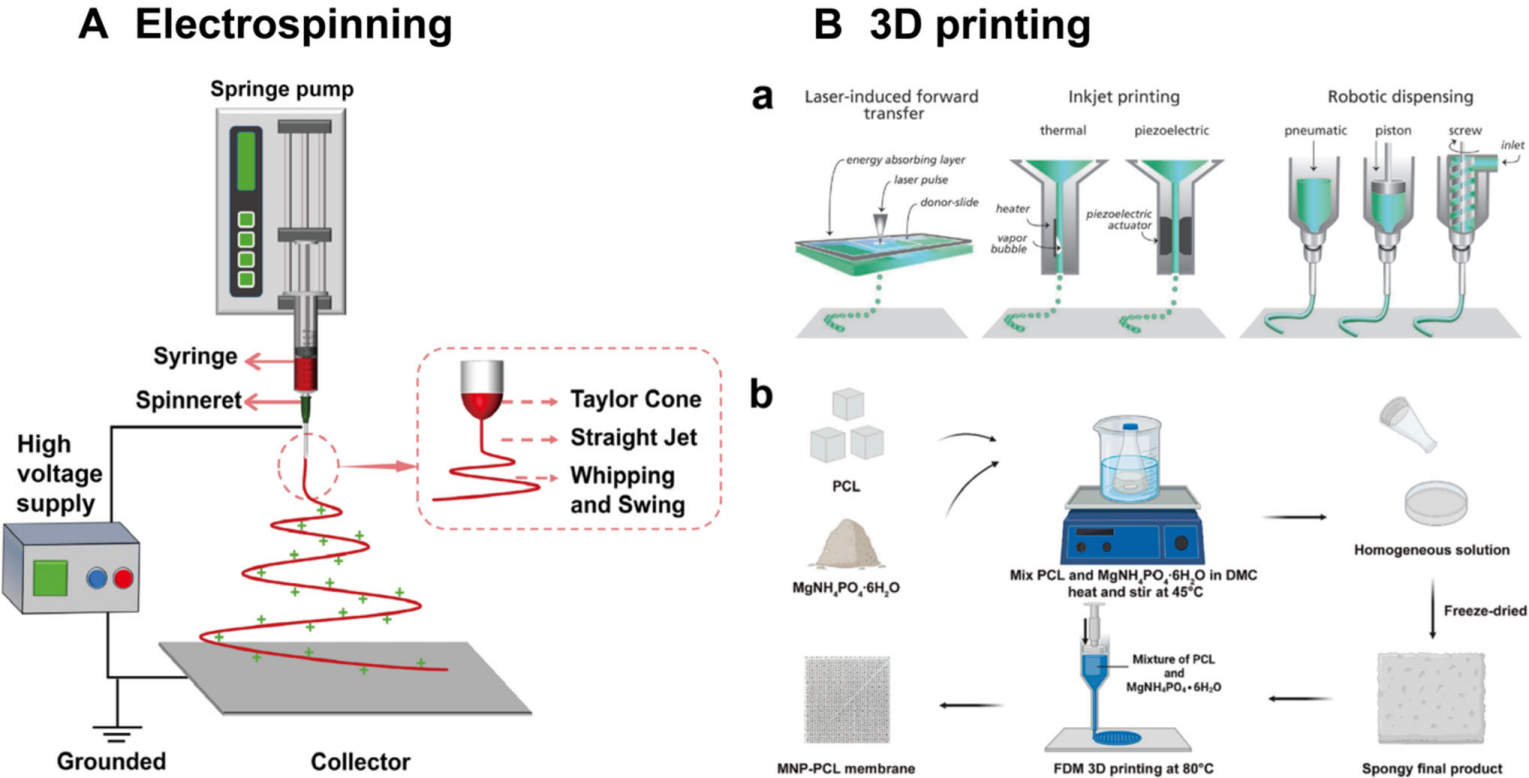
**A** The schematic diagram of the electrospinning process. Standard electrospinning equipment primarily consists of a syringe pump, a high-voltage power supply, a grounded collector, and a spinneret with a needle. The working principle is that the high-voltage power supply creates an electric field between the grounded collector and the injection needle. A hemispherical droplet of polymer solution forms at the tip of the needle as the material is progressively extruded. The repulsive electrostatic force overcomes the surface tension of the polymer solution, resulting in the formation of polymer jets and the splitting of droplets, which generate various micro- or nanofiber patterns after solvent vaporization. Adapted with permission from Zhao et al. [156] Copyright (2022), Dove Medical Press. **B** Selected 3D fabrication methods, including inkjet printing, light -assisted printing (e.g. laser-induced) and extrusion printing (including robotic dispensing) in section a. Adapted with permission from Malda et al.[165] Copyright (2013), Wiley. In section b, MgNH_4_PO_4_⋅6H_2_O (MNP) and PCL are utilized to prepare a novel MNP-PCL composite GBR membrane via the FDM 3D printing, which is an admirable method for the function design of the interconnected network structure with great distribution uniformity. Adapted with permission from Liu et al. [168] Copyright (2024), Elsevier

Among these traditional techniques, electrospinning technology garners primary attention due to its ability to produce nanofibrous membranes [150]. Membranes or scaffolds made of nanofibers possess an elevated surface-to-volume ratio, which can promote cell adhesion, migration, proliferation, and differentiation, as well as regulate the behavior and expression of stem cells [151–153]. Additionally, the electrospinning technique can mimic the morphology of the ECM, which facilitates the passage of oxygen and nutrients through the material due to the appropriate pore connectivity of the membrane [154]. Standard electrospinning equipment primarily consists of a syringe pump, a high-voltage power source, a grounded collector, and a spinneret with a needle, as illustrated in (Fig. 4A) [150, 155, 156]. The working principle of the electrospinning device involves complex electro-physical interactions between electrostatic forces and the surface of the polymer solution. In brief, the high-voltage power supply creates an electric field between the grounded collector and the injection needle [155]. A hemispherical droplet of polymer solution forms at the tip of the needle as the material is progressively extruded. This droplet accumulates excessive surface charge when exposed to a high-voltage electric field, causing mutual charge repulsion that elongates the polymer droplet into a conical shape known as "a Taylor cone". As the electric field strength increases to a certain critical value, the repulsive electrostatic force overcomes the surface tension of the polymer solution, resulting in the formation of polymer jets and the splitting of droplets, which generate various micro- or nanofiber patterns after solvent vaporization [157–159]. Moreover, the shape and surface topography of electrospun nanofibrous membranes can be modulated by adjusting processing parameters, allowing customization according to the specific features of defects [160]. However, a significant drawback of electrospun membranes is that residual solvent in the materials can produce cytotoxicity, negatively impacting cells if the solvent has not evaporated completely [161]. Furthermore, electrospinning technology faces challenges in achieving proper and reasonable cell infiltration in dental barrier membranes, which greatly restricts its application in biomedical engineering [162]. Therefore, addressing these challenges should be a priority in future research on electrospun membranes.

### Three‐dimensional (3D) printing technology

Dental tissues, such as alveolar bones and periodontal ligaments, exhibit complex spatial architectures, unique cell interactions, and anisotropic mechanical properties [163]. Despite significant advances in membrane fabrication, challenges remain in methods based on conventional techniques like electrospinning or injection molding. 3D printing, an additive manufacturing technology that fabricates objects by adding materials layer by layer to create a 3D volumetric structure [164], is driving major innovations in regenerative dental fields today. Recent studies have enabled it of both biocompatible materials and living cells to create complex functional tissues, a process known as "bioprinting." A key feature of bioprinting is that the deposition process must be cytocompatible, as it requires dispensing cell-containing media. This requirement limits the range of additive manufacturing techniques due to the need to maintain a proper environment at room temperature or 38 °C [164].

As is shown in Fig. 4B, the mainstream 3D printing methods include inkjet printing, extrusion, and light-assisted printing [163, 165]. Inkjet printing utilizes pressure pulses (thermal, acoustic, piezoelectric, etc.) to position biomaterials (primarily in liquid form) at specific locations, depositing them into designed structures [166]. However, the viscosity of biomaterials still poses a limitation. Higher viscosity droplets require excessive force for deposition, so it typically needs to be below 10 centipoises.

Extrusion printing, which uses mechanical extruders such as pneumatic, piston, and screw systems to continuously form materials, is most commonly used in dental membrane applications [167]. It can be divided into thermal and non-thermal processes. Among these, Fused Deposition Modeling (FDM) is a representative thermal extrusion 3D printing method (Fig. 4B) that shows promise for creating interconnected network structures with great distribution uniformity, based on layer-by-layer printing of thermoplastic polymers [168]. Compared to other methods, extrusion printing offers considerably better resolution, speed, spatial controllability, and greater flexibility in the finished membranes [169]. Nevertheless, it is limited in bioprinting systems where cells are encapsulated, as the extruding pressure and high temperatures in thermal extrusion printing can inevitably harm human cells [170].

Light-assisted 3D printing techniques, such as laser-assisted printing, photocuring, or stereolithography, rely on light polymerization of the biomaterial to generate printed products[171, 172]. These methods possess numerous advantages, including high resolution and great efficiency. However, challenges in light-assisted printing remain. For instance, photosensitive polymers or additives, which may not be biocompatible, are often required, restricting their application in tissue regeneration.

## Application for dental membranes

### Bone regeneration

**Fig. 5:**
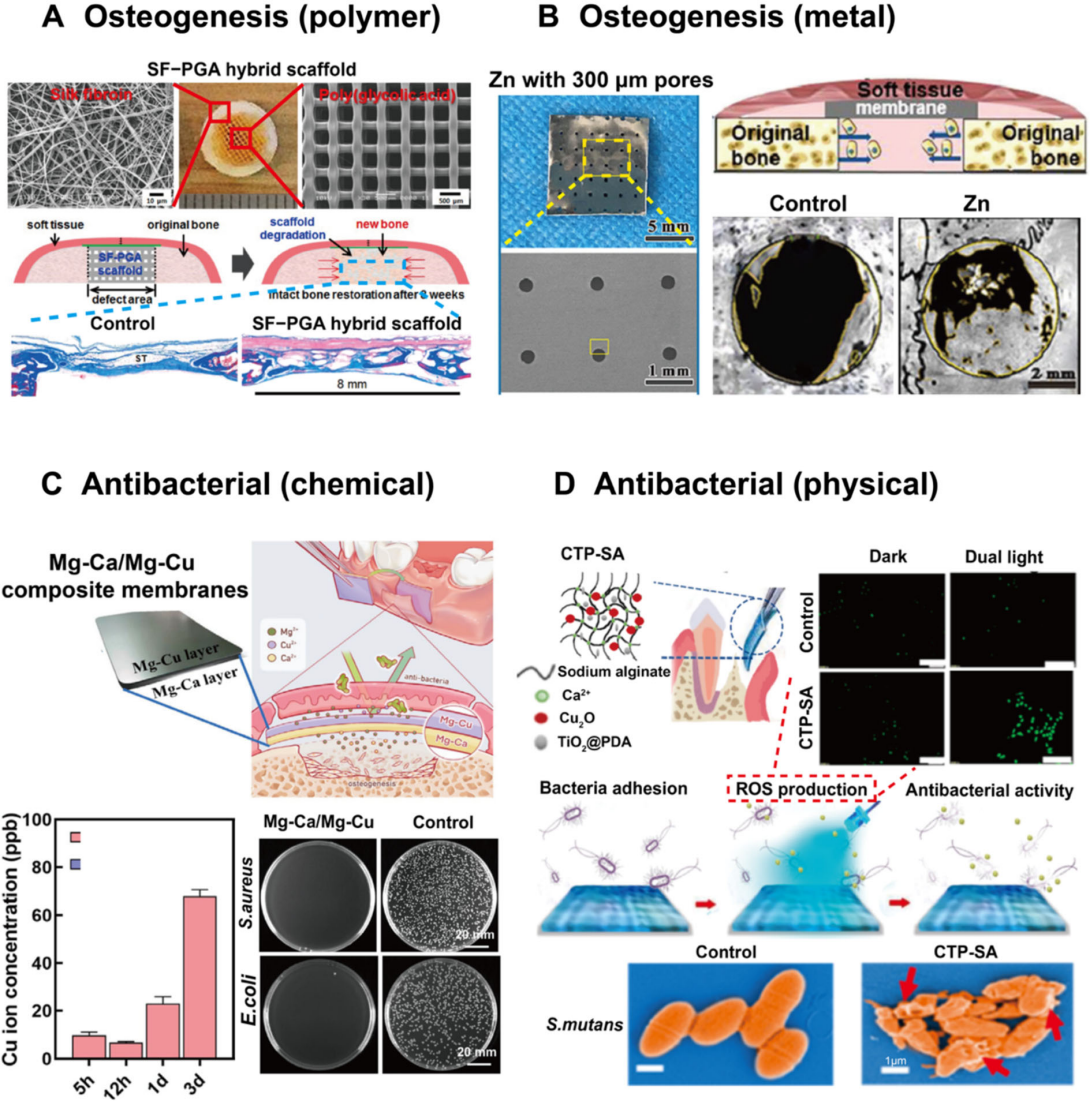
**A** Design of SF nanofiber membranes and poly (glycolic acid) scaffolds for osteogenesis. Adapted with permission from Kim et al. [180] Copyright (2019), American Chemical Society. **B** Pure Zn membrane with 300 μm pores displayed acceptable cell cytocompatibility in vitro and favorable osteogenic ability *in vivo*, demonstrating promising potential application in GBR membrane. Adapted with permission from Guo et al. [99, 105] Copyright (2020), Elsevier. **C** Design of Mg–Ca/Mg–Cu bilayer membranes by releasing Cu^2+^ ions for antibacterial property in GBR applications. Adapted with permission from Shan et al. [204] Copyright (2024), Elsevier. **D** Schematic diagram of the construction of sodium alginate hydrogel composite and its antibacterial performance by producing ROS under blue light irradiation. Adapted with permission from Xu et al. [207] Copyright (2020), American Chemical Society

GTR/GBR was initially developed to promote bone formation in defect areas, making osteogenesis one of its most important properties [173]. Numerous strategies have been devised to enhance its effectiveness, including structural modifications and the incorporation of biological cues. The physical properties of membranes can significantly influence cell adhesion, proliferation, and differentiation. For example, the topological structure of the membrane (e.g., a rough surface) can directly improve cell adhesion and spreading behavior. It has been reported that nanotubular topographies with diameters of 30 and 100 nm upregulate adhesive-related proteins and the (Wingless/ Integrated/beta-catenin)Wnt/β-catenin signaling pathway in osteoblast-like MG63 cells, which promotes osteogenesis [174], as well as tissue–material bonding in biomaterial–cell interactions [175]. Additionally, hydrophilic surfaces are more attractive to osteoblasts [176]. Therefore, the surfaces of membranes, particularly those made of bioinert synthetic polymers, are often tailored through acidic or alkaline treatments, anodic oxidation, or micro-patterning to promote bone formation, as illustrated in Fig. 5A [177–180].

Historically, many inorganic compounds, such as synthetic calcium phosphates, including HA, β-TCP, and their combination into biphasic calcium phosphates, have been commonly employed in orthopedic and dental settings due to their osteoinductive properties [181–183]. This category of synthetic ceramics facilitates vascular penetration, cellular infiltration and adhesion, cartilage formation, and the deposition of calcified tissue. Additionally, metallic inorganic compounds and their alloys are widely used in this field [184]. Mg and its alloys have demonstrated excellent biocompatibility and are commonly used in bone repair materials. Studies have shown that Mg^2+^ can enhance BMPs receptor recognition and integrin-mediated signaling cascades, stimulating the osteogenic differentiation of stem cells and playing a critical role in bone metabolism by promoting osteoblast proliferation and protecting against excessive bone resorption [185–188]. Zn (Fig. 5B) [99, 105] and strontium (Sr) exhibit similar osteogenic properties by continuously releasing ions when applied to bone defect areas, thereby achieving a balance between bone formation and resorption regulated by osteoblasts and osteoclasts [189–191]. However, their effectiveness can be influenced by the dose and the different release rates observed *in vivo* versus *in vitro*.

As mentioned earlier, numerous studies have revealed that cytokines and growth factors, such as BMP-2 [192], VEGF [193] and bone forming peptide-1 [194] have been widely utilized. However, some studies indicate that higher doses may impede broader use due to the potential for inflammatory responses.

### Antibacterial property

GTR/GBR is often placed in specific open incision areas for implantation or periodontal surgery, where injured tissues and inflammatory cells accumulate [195]. Additionally, because the oral cavity is directly exposed to the external environment, the microbially rich oral surroundings present significant challenges for dental membrane applications [196]. Postoperative infections due to bacteria are common complications after surgeries, leading to delayed wound healing and inhibited bone regeneration, and even surgical failure [197]. Given these issues, there is an urgent need for the development of barrier membranes with bacterial inhibition capabilities.

Antibacterial strategies in dental membranes can typically be categorized into chemical and physical methods. Traditionally, commercial chemical drugs such as metronidazole, amoxicillin, and ammonium chloride have been loaded into dental membranes for sterilization [198–200]. These drugs inhibit bacterial deoxyribonucleic acid (DNA) synthesis and metabolism. Despite their prominent antibacterial properties, these drugs have gradually been replaced due to side effects, such as the development of bacterial strain resistance. Recently, researchers have incorporated metals such as copper (Cu), Ag, and Zn ions (Fig. 5C), as well as their composites, into membranes to enhance antimicrobial properties [201–204]. These metals can kill bacteria by producing reactive oxygen species (ROS), such as singlet oxygen (^1^O_2_), superoxide (O_2_^.-^), hydrogen peroxide (H_2_O_2_), and hydroxyl radicals (·OH) [205, 206]. They can also penetrate bacterial cell membranes, affecting permeability and interacting with bioactive proteases, ultimately disrupting DNA replication [204]. Moreover, these metals and their composites can help avoid drug resistance and may even accelerate bone regeneration (e.g., Mg and Zn). However, potential cytotoxicity, especially to mammalian cells, cannot be ignored, even when metal concentrations are adjusted for biocompatibility. Furthermore, the limited release time and uncontrolled release rate remain significant restrictions for all chemical antibacterial strategies.

To address these challenges, photodynamic therapy (PDT) and photothermal therapy (PTT) have emerged as prominent physical sterilization strategies widely used in GBR today. As it is shown in Fig. 5D, by irradiating photosensitizers assembled on the surface of dental membranes with specific wavelength light, PSs absorb light energy and react with ambient oxygen to produce ROS, leading to bacterial damage and death [207]. Additionally, some high photothermal conversion materials (such as MoS_2_) are loaded onto membranes to achieve temperatures above 45 °C under light irradiation in PTT [208, 209]. In such high-temperature environments, bacteria are inactivated, resulting in the destruction of their spatial structure and function, ultimately leading to bacterial death [210]. Although PDT and PTT provide direct and effective antibacterial effects, they also have unavoidable drawbacks. First, the ROS generated in PDT have a short half-life and limited diffusion radius, which may hinder their antibacterial effectiveness. Furthermore, the high temperatures involved in PTT can inevitably damage the surrounding periodontal tissue. Thus, maintaining a balance between tissue induction and bacterial inhibition remains a challenge.

### Mechanical support

Severe bone defects in the maxilla and mandible caused by tooth loss, trauma, or tumors often lead to the deterioration of the original bone dimensions [211]. Given this demand, GTR/GBR membranes should be designed to prevent collapse and preserve bone volume, which is essential for facilitating subsequent plastic surgery or the placement of dental implants.

Generally, non-resorbable membranes, primarily Ti mesh and PTFE, exhibit favorable mechanical stiffness and provide high volume stability [212, 213]. Furthermore, studies have shown that Ti reinforcement of high-density PTFE membranes enhances regenerative capacity compared to traditional expanded PTFE membranes [214]. This improvement is mainly due to the additional mechanical support provided by the Ti frame against the compressive forces exerted by overlying soft tissue [214]. However, the lack of biodegradability necessitates a secondary surgical procedure for removal [215, 216]. Additionally, excessive membrane stiffness may cause soft tissue dehiscence, increasing the likelihood of wound infection and extending the healing period [217–219].

In response to these challenges, resorbable membranes made from natural collagen, synthetic polymers, and metals have been developed. Synthetic polymers are biocompatible and biodegradable. However, the poor mechanical support properties of both natural and synthetic membranes are significant limiting factors due to insufficient compressive strength. Although these membranes can demonstrate high strength initially, they often completely lose their mechanical properties within four weeks of incubation in culture medium [220–222]. Consequently, various strategies, including modifications to fabrication methods and specific structural designs, have been implemented to inhibit membrane collapse. It has been reported that the stiffness and degradation time can be increased by incorporating certain (in)organic particles, such as Sr apatite [223], amorphous calcium [224], tricalcium phosphate [225]. Moreover, manufacturing methods like electrospinning for specific structures (e.g. bilayers), cross-linking using ultraviolet light or glutaraldehyde plus irradiation, and 3D printing to model membrane porosity can also enhance membrane durability and boost biomechanical properties.

**Fig. 6:**
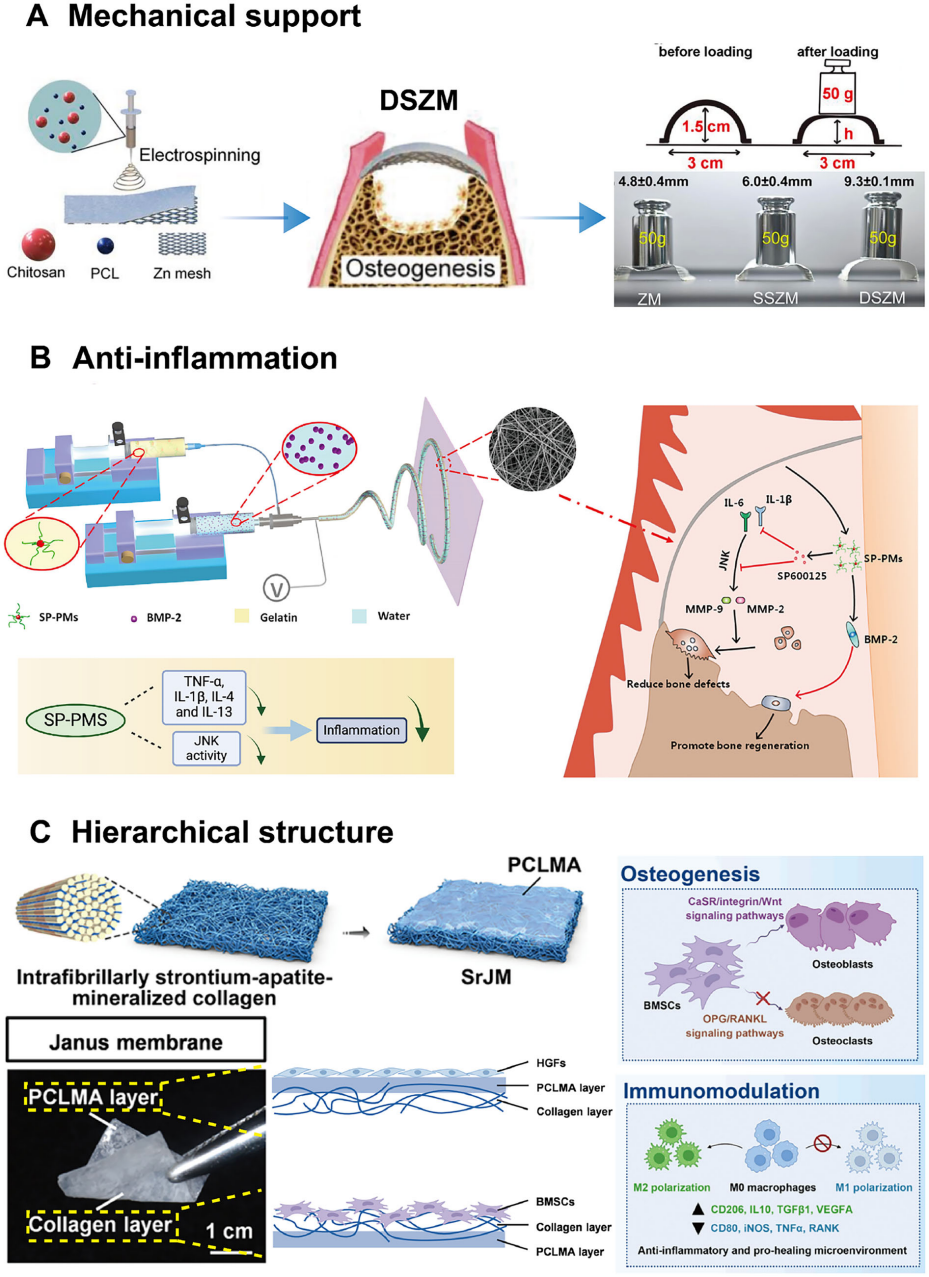
**A** Fabrication, function and enhanced mechanical property of polycaprolactone and chitosan layers on a Zn mesh surface for promising use in support defect areas. Adapted with permission from Xu et al. [226] Copyright (2024), Elsevier. **B** Design and anti-inflammatory mechanism of a core–shell micelle-in-nanofiber membrane by carrying SP600125-loaded polymeric micelles, which could downregulate the inflammatory factors such as Interleukin-4(L-4) and Tumor Necrosis Factor-Alpha(TNF-α), resulting in decreased inflammation. Adapted with permission from Liu et al. [234] Copyright (2020), Elsevier. **C** A Janus membrane named SrJM is developed, the hierarchical structure of which consists of a porous collagen face (strontium-apatite-mineralized collagen) to enhance osteogenesis and immunomodulation and a dense face (polycaprolactone methacryloyl) to maintain barrier function. Adapted with permission from Zhao et al. [223] Copyright (2024), American Chemical Society

To further optimize the support performance and biodegradability of barrier membranes, metal-based materials, such as those made from Zn (Fig. 6A) and Mg, have emerged as focal points for GBR applications in recent years as discussed above [204, 226]. However, the degradation rate of metal alloy membranes should be accurately adjusted to align with the periodontal regeneration timeline.

### Anti-inflammation

Periodontitis and trauma are the major application fields for GTR/GBR membranes, where the periodontium or craniomaxillofacial bone suffers from inflammation due to bacteria or excessive host immune response. Accordingly, an ideal dental membrane should possess the following characteristics: I) Biocompatibility to allow integration with host tissues without eliciting a foreign body response and II) Anti-inflammatory properties to promote wound healing and tissue regeneration [227].

Generally, the composition of the membrane often influences its immunomodulatory features. To begin with, compared to absorbable membranes, non-degradable materials typically provoke the formation of fibrotic capsules, which can lead to foreign body reactions [228]. Additionally, the origin of the material (fossil-based or bio-based polymers) also modulates the immune response. Unlike synthetic polymer membranes (e.g., PCL), natural membranes tend to interact better with the host immune system due to their homology and comparatively weaker mechanical support, which helps avoid wound dehiscence and other postoperative adverse reactions [229]. Recently, many strategies have been proposed to minimize inflammation. First, the topography and micro-architecture of the membranes—characterized by porosity, hydrophobicity, stiffness—are critical in influencing inflammation [230, 231]. For example, several studies have shown that the diameter and organization of the membrane structure affect macrophage polarization and the degree of the inflammatory response. Materials with larger pore sizes are more favorable for macrophages 2(M2) polarization of immune cells, which contributes to inflammation reduction [232, 233]. However, it is also crucial to maintain a balance, as multiple large pores might negatively affect the mechanical stability of the structure.

Furthermore, many biomaterials and drugs with ability to adjust the inflammatory factors (Fig. 6B) have been incorporated into the membranes [234]. Non-steroidal anti-inflammatory drugs, such as ibuprofen and piroxicam, inhibit the activity of cyclooxygenase, thereby preventing the conversion of arachidonic acid into prostaglandins which can exert negative effects on periodontal regeneration [235, 236].

### Biomimetic and hierarchical structure

Tissue engineering scaffolds with specific surface morphologies play a crucial role in regulating cellular behaviors and facilitating tissue repair processes [237, 238]. Among these surface structures, biomimetic surfaces, such as triply periodic minimal surfaces (TPMS), and intelligent hierarchical structures like Janus membranes have garnered significant attention for their potential in membrane applications [239, 240].

TPMS, characterized by infinite duplication in three dimensions and zero mean curvature, offer a unique gradient scaffold design with radially graded pores [241, 242]. These pores can be tailored to have varying diameters that align with the specific requirements of bone tissue engineering. The porous nature of TPMS, devoid of sharp turns or junction points, enables them to effectively withstand compressive loads, exhibiting energy absorption properties and serving as efficient energy buffers during mechanical stresses [241, 243]. On the other hand, Janus membranes, featuring a bilayer structure with distinct surface properties on each side, have been explored for their ability to promote osteogenic functions while maintaining a barrier function for gingival fibroblasts (Fig. 6C) [223]. Lv et al*.* developed a Janus carboxymethyl chitin/HA porous membrane for applications in hemostasis and osteogenesis [239]. In their study, they incorporated the soluble salt NaH_2_PO_4_ into the carboxymethyl chitin solution as a porogen. The top layer of the membrane is designed to prevent epithelial cell infiltration, exhibiting a relatively non-porous structure, while the base layer consists of pores ranging from 100 to 200 μm to facilitate the proliferation of osteoblasts [244].

### Other applications for GBR

**Fig. 7:**
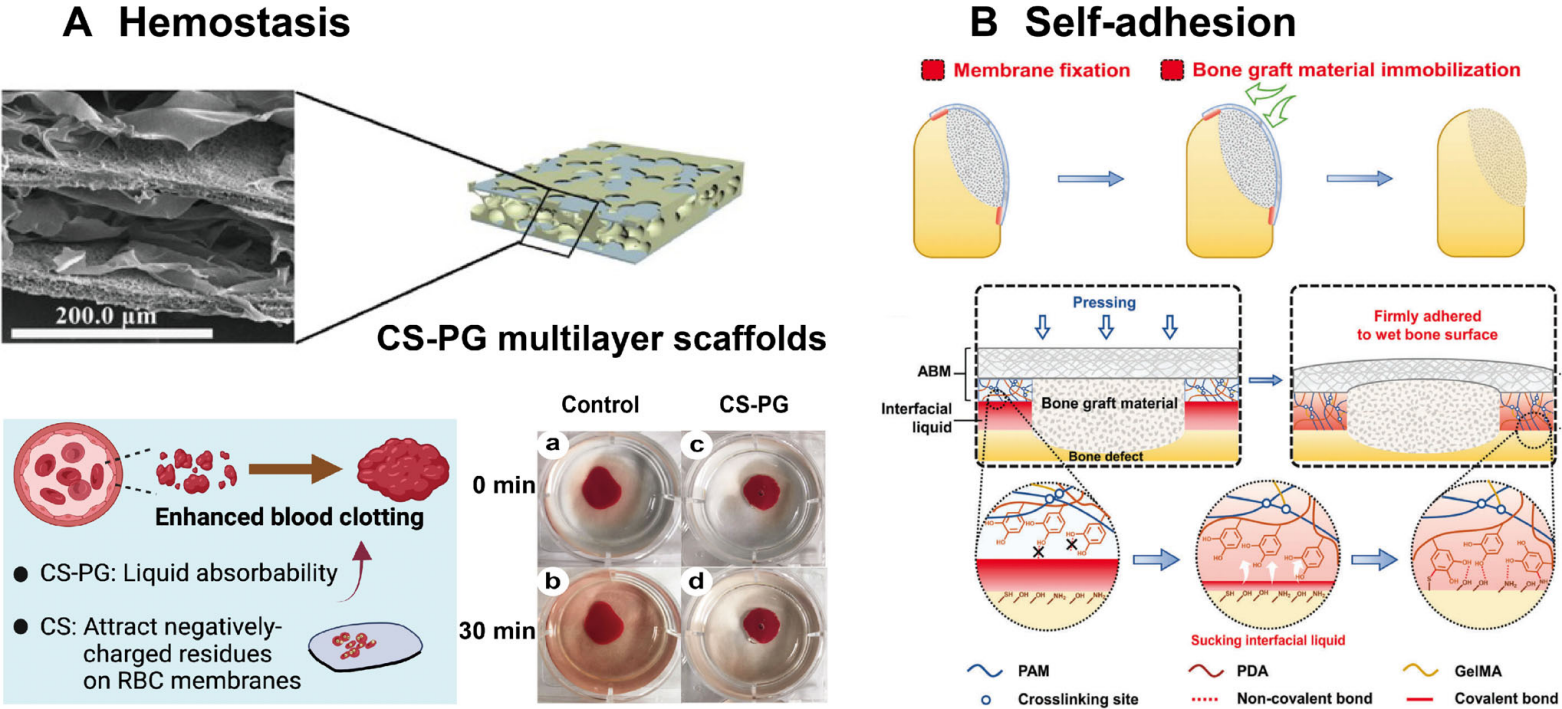
**A** Design and mechanism of composite materials of CS with Polycaprolactone and Gelatin multilayer scaffolds for effective hemostasis. CS could improve liquid absorbability and gather the blood cells. Adapted with permission from Zhang et al. [247] Copyright (2020), Elsevier. (Created with https://BioRender.com) **B** Ingredients, application and mechanism of a band-aid-like self-fixed barrier membrane contributing to superior bone augmentation. Once pressed to the bone defect areas, the self-fixed barrier membrane firmly adheres to wet bone surface without movement. Adapted with permission from Li et al. [245] Copyright (2023), Wiley

Even though dental barrier membranes were initially developed to exclude non-osteogenic tissues from influencing the bone healing process, there are now numerous types with customizable physicochemical properties for preclinical applications such as hemostasis [239] and self-adhesion [245]. Bone defects or implantation surgeries often lead to bone hemorrhage, which can hinder surgical procedures and cause serious postoperative complications. Therefore, accelerating blood clotting and achieving effective hemostasis is vital for initial recovery [246]. However, most GBR membranes can only restrict blood accumulation beneath the membrane, lacking practical hemostatic properties. Zhang et al*.* fabricated multifunctional scaffolds that provide hemostatic and cell barrier functions, utilizing CS as a styptic agent. CS forms cationic clusters that interact with anions on red blood cells and activate platelets [247]. Additionally, the porosity and water absorbance properties of hierarchical pore composite scaffolds enhance the adhesion of more blood cells, as illustrated in Fig. 7A.

Self-adhesion is another intriguing and promising property for GBR applications. During the wound closure and healing process, external forces on the augmented bone area—such as those generated during flap closure, the movement of adjacent muscles, and chewing—can cause significant displacement of barrier membranes, adversely affecting the quality of bone augmentation [248, 249]. Therefore, it is crucial to maintain the GBR in the desired position. A new class of local adhesive barrier membranes was developed by Li et al*.* to immobilize bone graft materials (Fig. 7B) [245]. These novel air-dried adhesive hydrogel layers, made from polyacrylamide/polydopamine, allow membranes to firmly adhere to wet bone surfaces through a "stick-and-use" band-aid-like strategy [250, 251]. Upon contact with moisture on the bone surface, the hydrogels quickly swell and become flexible, enabling them to adapt to various irregular bone surfaces and establish a continuous, seamless barrier for bone grafts, effectively preventing membrane displacement and the leakage of graft materials.

## Summary and outlook

To date, barrier membranes are not only a promising clinical method for shielding defect areas from soft tissue ingrowth but are also widely used in antibacterial, anti-inflammatory, and other functional applications. Hence, this review focuses on the fundamental aspects of materials and manufacturing methods, providing the latest ideas on how to design functional membranes for the entire tissue regeneration process. Additionally, the strengths and weaknesses are directly presented, paving the way for further advancements in the field of dental membranes.

Despite these encouraging improvements in barrier membranes, several issues remain to be addressed. Firstly, to the best of our knowledge in the field of periodontology, the majority of current research on membrane modification focuses solely on osteogenesis, neglecting the fact that ideal healing involves both periodontal ligament reconstruction and osteogenesis. Given that the periodontal ligament provides buffering, nutrition, and sensory functions for surrounding tissues but is challenging to repair [252], future research should prioritize this area. Secondly, regarding material selection and improvement, there is often a trade-off between membrane stiffness and biodegradability [253]. Therefore, research into developing membranes that combine proper mechanical support with biomimetic degradability is of great significance. Moreover, since degradation is a dynamic process, the inevitable decline in mechanical support and reduction of the shielding effect should also be considered. Accordingly, more emphasis should be placed on mechanical testing in *in vivo* experiments aimed at monitoring the entire dynamic process.

Additionally, it is acknowledged that tissue reconstruction after GBR/GTR surgery is a complex process that includes macrophage polarization, neutrophil recruitment, angiogenesis, and subsequently, osteogenesis [254]. More attention should be paid to utilizing bioactive agents (e.g., cytokines and antibiotics) and optimizing membrane morphology to dynamically modulate different healing stages and defect positions, thereby programming the entire regeneration process for enhanced regenerative effects.

To address these challenges, interdisciplinary collaboration among material scientists, bioengineers, and clinicians is urgently needed. This collaboration will not only shed light on functional periodontium and bone tissue regeneration but also accelerate the translation of research into clinical applications for public oral health.

## Data Availability

The datasets generated during and/or analyzed during the current study are available from the corresponding author upon reasonable request.
